# Eating patterns in relation to anthropometrics and blood pressure among adults with overweight and obesity – a cross-sectional study

**DOI:** 10.48101/ujms.v130.12227

**Published:** 2025-07-14

**Authors:** Elin Siurua, Kjell-Åke Alle, Mari Bergenholtz, Lena Lendahls, Sara Holmberg

**Affiliations:** a Kvinnokliniken, Vrinnevisjukhuset, Norrköping, Sweden; b Department of Research and Development, Region Kronoberg, Växjö, Sweden; c Faculty of Health Sciences; Linköping University, Linköping, Sweden; d Vårdcentralen Läkarhuset, Ljungby, Sweden; e Department of Medicine and Optometry, Faculty of Health and Life Sciences, Linnaeus University, Kalmar, Sweden; f Division of Occupational and Environmental Medicine, Department of Laboratory Medicine, Lund University, Lund, Sweden

**Keywords:** Eating habits, food intake, meals, weight, body mass index, waist circumference, hypertension

## Abstract

**Background:**

This study aimed to describe eating patterns among individuals with overweight and obesity and to investigate associations between eating patterns and anthropometric measures, including body mass index (BMI) and waist circumference, and blood pressure.

**Methods:**

This study enrolled a cohort of adults with overweight or obesity (*n* = 176) participating in a clinical trial focused on weight reduction. Self-reported eating patterns were assessed as part of the trial’s baseline survey. Trained study nurses conducted measurements of anthropometric indicators and blood pressure. To examine associations, statistical analyses included the application of the Mann-Whitney U-test, Fisher’s exact test, the chi-squared test, and linear regression models as appropriate.

**Results:**

The median age of the participants was 55 years (interquartile range [IQR] 12), 79% were female, and the median BMI was 33 kg/m^2^ (IQR 5). The predominant eating pattern identified was characterized by five meals per day, including breakfast, two prepared meals, and two snacks. Among older participants (≥ 55 years), 51% reported eating two prepared meals per day as compared to 75% among the younger (*P* <> 0.05). A higher percentage of older participants reported consuming more than one snack per day (82% vs. 68%, *P* = 0.04). Additionally, older participants were more likely to rate their eating habits as ‘good’ compared to their younger counterparts (64% vs. 52%, *P* = 0.03). Women reported a higher number of eating occasions than men (> 3/day: 93% vs. 78%, *P* = 0.01) and a higher frequency of snacks (> 1 snack/day: 79% vs. 61%, *P* = 0.03). No significant associations between the number of eating occasions or number of snacks and BMI, waist circumference, or blood pressure (systolic and/or diastolic) were found in regression models when age and sex were considered.

**Conclusions:**

Varying eating patterns were observed among adults with overweight and obesity according to age and sex. No association between eating patterns and anthropometric measures or blood pressure independent of age and sex was found.

## Introduction

The global rise in overweight (body mass index [BMI] ≥ 25 to < 30 kg/m^2^) and obesity (BMI ≥ 30 kg/m^2^) poses a significant health challenge (1), with 39% of the Swedish adult population being overweight and 12% having obesity in 2022 (2). Obesity management is costly and contributes to escalating healthcare expenses. Diet plays a pivotal role in weight management, alongside other health behaviors. Beyond macronutrient composition and diet quality, aspects of eating behavior, such as meal timing, size, frequency, and breakfast consumption, influence health and weight control. ‘Eating patterns’ describe the distribution of eating occasions and size of meals over a day or over longer periods, while ‘eating habits’ often also encompass diets, food type, and nutritional content. Eating patterns have been linked to weight and health outcomes (3–6), but less is known compared to what is known about associations between diet and health.

Increased eating frequency has been suggested as a strategy for weight loss and maintaining a healthy weight (6). Some studies indicate that more eating occasions enhance appetite control and reduce obesity risk (6, 7), but recent larger studies have linked more frequent eating occasions to weight gain (4, 5). A 2017 study involving over 50,000 healthy adults in the US and Canada explored meal frequency and timing, revealing that consuming fewer meals, no snacking, eating breakfast, and having the largest meal at breakfast could help prevent weight gain over a mean follow-up of 7 years (5), although its generalizability was limited to members of a specific church. Benefits of breakfast consumption include improved weight control, reduced risk of type 2 diabetes, and lower coronary heart disease risk (4–6, 8, 9). However, conflicting data question breakfast’s health value (10). The relationship between eating patterns, meal timing, meal frequency, and body weight is increasingly studied, but consensus on the preference for increased or reduced meal frequency in better weight management remains elusive (4–7).

Additionally, waist circumference complements BMI for metabolic assessments in clinical settings and might predict metabolic health risks better than BMI (11). Dietary intake patterns have been found to be similarily associated with BMI and waist circumference (12, 13), but there appears to be limited research.

Research into potential associations between eating patterns and blood pressure is limited despite the established connection between obesity and hypertension (14). High blood pressure is one of the components of the metabolic syndrome. Dietary intake, such as adherence to the DASH diet, has been found to reduce the risk of hypertension (15). An Australian study in 2019 reported an inverse link between snack frequency and blood pressure in men, which disappeared after adjusting for other dietary factors (16). A ‘later lunch’ pattern was associated with higher blood pressure in women in the same study.

Scientific uncertainties persist regarding the optimal diet for health and dietary advice, with significant knowledge gaps remaining concerning eating patterns and health-related outcomes.

The objectives of this cross-sectional study were twofold: first, to describe and provide a detailed characterization of eating patterns among adults with overweight and obesity participating in an intervention study, and second, to investigate associations between eating patterns and anthropometric measurements, and blood pressure, and self-reported evaluations of eating habits among the participants.

## Materials and methods

### Design

Baseline data from a randomized controlled trial testing an intervention for weight loss were used for cross-sectional analysis. The trial intervention was an educative group concept for weight loss (named ‘Dare to feel full’; ‘Våga vara mätt’ in Swedish), focusing on eating for satiety and normal weight, food content and metabolic effects on blood sugar related to various foods, and how to have a healthy relationship with food (17).

### Subjects

Participants were recruited from the public in two neighboring rural counties with a total population of about 450,000 inhabitants in southern Sweden via media advertisements and websites. Interested individuals underwent telephone interviews conducted by a project nurse, applying a predefined protocol outlining inclusion and exclusion criteria. Exclusion criteria were multiple food allergy, insulin-treated diabetes, severe mental illness, severe liver or kidney disease, heart failure grades 3–4, or other significant general medical conditions. Two hundred (*n* = 200) Swedish speaking residents aged 18–70 years with a BMI of 27–45 kg/m^2^ were enrolled in the study, half from each of the counties. However, 24 individuals did not attend the initial encounter, resulting in a final baseline investigation cohort of 176 participants.

### Data collection

All participants filled in a survey questionnaire, developed for the study, at a baseline health examination. The questionnaire was based on established items, about lifestyle, eating habits, quality of life, and health and illness (Supplementary 1) (17). Height, weight, and waist measurements were taken by a study nurse. Systolic and diastolic blood pressures were taken using a manual blood pressure monitor after sitting rest. Two measurements were performed in the right arm a few minutes apart, and the mean value was noted.

Eating patterns were assessed by an open matrix question where participants were asked to fill in everything they eat and drink on a typical weekday, which was split into six different ‘windows’ of eating: early morning, late morning, midday, afternoon, evening, and night. The participants were asked to write down everything they ate within each of these eating windows. Self-rated eating habits were assessed by a multiple-choice question with four answer options: ‘Very good’, ‘Good’, ‘Bad’, and ‘Very bad’. The questions were answered on paper forms and were later coded and transferred into a database by the first author.

### Categorization of eating pattern

Breakfast, a prepared lunch, and a prepared dinner were considered main meals, while eating in between meals or replacing meals with unprepared food was considered snacking. Coded variables were as follows: the number of eating occasions, timing of the largest meal, consumption of breakfast, and the number of prepared meals and snacks. Eating within either of the first two eating-windows (consuming food before midday) was assessed as breakfast. The number of meals was counted by summarizing the number of eating windows in which food had been consumed. One eating window could include two separate eating occasions. An eating window was coded as two separate eating occasions if the food consumed was noted far apart from one another within the window or if the participant specified the time of two different eating occasions. If the food-components of the main meals (lunch/dinner) were not specified and instead documented as either ‘lunch’ or ‘dinner’ by the participant, it was registered as a prepared meal. If options of both prepared food and unprepared food were given by the participant in one eating window, the first option given was considered the most common and was therefore registered. Eating a pre-packaged meal or eating out was classified as a prepared meal. As many participants wrote the time of the food being consumed, the timing of the meal was used to classify the type of main meal being consumed. In these cases, lunch was assessed as a prepared meal consumed between 12:00 and 15:00, and dinner was considered being a prepared meal consumed between 15:00 and 21:00.

For a meal to be considered as a main meal (lunch or dinner), it had to include prepared foods and be consumed within the time-windows previously mentioned. If consuming prepared foods at both lunch and dinner time, the main meals were considered similar in size. A prepared meal was always considered as being a larger meal than an unprepared meal. Any eating occasion that was not breakfast or classified as a main meal was considered a snack.

### Statistical analysis

All statistical analyses were performed with SPSS 27. A power analysis was performed prior to the intervention trial, which aimed to compare weight changes between two study groups, showing that 200 participants were needed to achieve a 80% power at alfa 0.05 (G*Power 3.1) (17). No power calculation was performed for the baseline cross-sectional analysis.

Categorial data were reported as number (*n*) and proportion (%), and continuous data were reported as median with interquartile range (IQR), as the Shapiro-Wilk test for normality indicated that the data were not normally distributed. Self-rated eating habits were recoded into a binary variable with only two categories: ‘Good’ or ‘Bad’. The Mann-Whitney U-test was used to compare different sets of eating patterns to BMI, waist circumference, and systolic and diastolic blood pressures. The Fisher’s exact test and chi-square test were used to compare eating patterns between age groups, between men and women, and regarding self-rated eating habits. Linear regression models were constructed to evaluate the association between various eating patterns (independent variables) and anthropometric measurements or blood pressure (dependent variables). The models were adjusted for age and sex. A *P*-value < 0.05 was considered significant.

### Ethical considerations

The intervention study was approved by the Ethics Review Board in Linköping, Sweden, Dnr: 2014/231-31. All participants signed a written informed consent.

## Results

One hundred and seventy-six subjects participated in the baseline survey, which was 88% of the randomized participants (Table 1). There were more female participants than males (79% vs. 21%). The most common eating pattern was consuming food five times per day. This included eating breakfast followed by snack later in the morning. The food intake at lunch and dinner time mainly consisted of prepared meals. Consuming a second snack in the afternoon was also a part of the most typical eating pattern. Furthermore, almost a quarter of the participants consumed some sort of snack later in the evening after having dinner. All but two participants consumed breakfast in a typical day. A majority of participants rated their eating habits as being ‘Good’.

**Table 1:** Characteristics of the study population (*n* = 176).

Variables	Median (IQR)	Number (%)
**Age (years)**	55.5 (12.0)	
**Sex (male/female)**		36/140 (21/79)
**BMI (kg/m^2^)**	32.9 (5.3)	
**Waist circumference (cm)**	104.0 (13.0)	
**Blood pressure (mmHg)**		
Systolic	135.0 (10.5)	
Diastolic	80.0 (5.0)	
**Number of eating occasions/day**	5.0 (1.0)	
**Eating occasions/day, specified**		
2		1 (1)
3		17 (10)
4		54 (31)
5		72 (41)
6		32 (18)
**Number of snacks/day^1^**	2.0 (1.0)	
**Snacks/day, specified**		
0		12 (7)
1		31 (18)
2		75 (43)
3		51 (29)
4		7 (4)
**Number of meals with prepared food/day**	2.0 (1.0)	
**Prepared meals/day, specified**		
0		1 (1)
1		65 (37)
2		110 (63)
**Consumption of breakfast (yes/no)**		174/2 (99/1)
**Largest meal consumed/day**		
Lunch		50 (28)
Dinner		29 (17)
Two large meals		96 (55)
No prepared meal		1 (1)
**Self-reported eating habits**		
Very good		1(1)
Good		102 (60)
Bad		55 (33)
Very bad		11 (7)

IQR: interquartile range; BMI: body mass index.

1 A snack is defined as eating in between meals or replacing meals with unprepared food.

Eating patterns by the total number of eating occasions per day, the total number of snacks consumed per day, the total number of meals with prepared food consumed per day, and self-rated eating habits in relation to anthropometrics and blood pressure are shown in Table 2. No significant associations between number of eating occasions or number of snacks and BMI, waist circumference, or blood pressure (systolic and/or diastolic) were found when age and sex were considered (Table 2).

**Table 2:** Eating patterns and self-rated eating habits in relation to anthropometrics and blood pressure (median [IQR]).

	≤ 3 meals/day	> 3 meals/day	*P* ^1^	0–1 snack^2^/day	> 1 snack/day	*P* ^1^	1 prepared meal/day	2 prepared meals/day	*P* ^1^	Good	Bad	*P* ^1^
***n***	18	158		43	133		65	110		103	66	
**BMI (kg/m^2^)**	33 (8)	33 (5)	0.92	33 (8)	33 (5)	0.53	33 (6)	33 (5)	0.42	33 (6)	33 (5)	0.42
**Waist (cm)**	109 (15)	104 (12)	0.37	107 (16)	103 (12)	0.10	104 (11)	104 (14)	0.66	104 (15)	104 (12)	0.99
**Blood pressure (mm Hg)**												
Systolic	140 (19)	135 (10)	0.31	130 (11)	135 (11)	0.80	140 (16)	130 (15)	**0.03^3^**	135 (19)	130 (10)	0.57
Diastolic	88 (10)	80 (5)	0.18	84 (10)	80 (5)	0.50	80 (10)	80 (5)	0.61	80 (5)	85 (10)	0.06

BMI: body mass index.

1 *P*-values for differences within the four groups (meals/day, snacks/day, prepared meals/day, and good/bad) were calculated using Mann-Whitney U-test with a *P*-value < 0.05 considered significant (bold).

2 A snack is defined as eating in between meals or replacing meals with unprepared food.

3 No significant association when age and sex were considered (linear regression).

A significant difference in eating patterns according to age was seen on all patterns except total number of eating occasions per day where no difference was detected. A larger proportion in the older age group (> 55 years) consumed more than one snack per day, and fewer ate two prepared meals per day. Participants < 55 years of age were more likely to consume two prepared meals per day and less likely to snack than the older age group. Participants in the older age group rated their eating habits as ‘Good’ to a greater extent than the younger group (64% vs. 52%, *P* = 0.03). A higher proportion of female participants ate more often than three times per day, as compared to men (93% vs. 78%, *P* = 0.01) (Table 3). Also, a larger proportion of females ate more than one snack per day (79% vs. 61%, *P* = 0.03). No significant relationships between self-rated eating habits and eating patterns were found.

**Table 3:** Participants eating patterns in relation to sex, age, and self-rated eating habits.

Variables^1^	Female	Male	P^2^	Age < 55 year	Age ≥ 55 year	P^2^	Good eating habits	Bad eating habits	P^2^
***n* (%)**	140 (79)	36 (21)		81 (46)	95 (54)				
**Number of eating occasions/day**									
≤ 3	10 (7)	8 (22)	**0.01**	11 (14)	7 (7)	0.21	10 (10)	7 (10)	1.00
> 3	129 (93)	28 (78)		70 (86)	87 (93)		93 (90)	59 (90)	
**Number of snacks/day^3^**									
0–1	29 (21)	14 (39)	**0.03**	26 (32)	17 (18)	**0.04**	28 (27)	14 (21)	0.47
> 1	110 (79)	22 (61)		55 (68)	77 (82)		75 (73)	52 (79)	
**Number of meals with prepared food/day**									
1	52 (37)	12 (33)	0.70	19 (24)	46 (49)	**0.00**	33 (32)	27 (41)	0.25
2	86 (62)	24 (67)		61 (75)	48 (51)		70 (68)	38 (58)	
**Largest meal consumed/day**									
Lunch	39 (28)	10 (28)	0.96	15 (19)	35 (37)	**0.04**	27 (16)	19 (11)	0.75
Dinner	24 (17)	5 (14)		9 (11)	19 (20)		16 (10)	12 (7)	
Two large meals	75 (54)	21 (58)		56 (70)	40 (43)		60 (36)	34 (20)	
**Self-reported eating habits**									
Good	82 (61)	20 (59)	0.85	42 (52)	60 (64)	**0.03**			
Bad	52 (39)	14 (41)		39 (48)	27 (29)				

1 All values are *n* (%) unless otherwise indicated.

2 Statistical analysis made using Fisher’s exact test (Chi-square test for the 3 × 2 tables) with a *P*-value < 0.05 considered significant (bold).

3 A snack is defined as eating in between meals or replacing meals with unprepared food.

In linear regression analyses with BMI, waist circumference, and systolic and diastolic blood pressures as dependent variables, no associations were found with the different eating patterns when adjusted for age and sex (Supplementary 2). Age and sex were as expected associated with anthropometrics and systolic blood pressure, but no relationship with diastolic blood pressure was seen.

## Discussion

This study investigated the eating patterns of a culturally homogenous group of Swedish adults with overweight and obesity, mainly women, participating in a weight reduction trial. The most common eating pattern was consuming food five times per day, including breakfast, two main meals (lunch and dinner), and two snacks with some differences in relation to age and sex. There were no associations between eating patterns and anthropometric measures or blood pressure after adjustment for age and sex.

No relationship between BMI and different eating patterns was seen in our study. Older studies have indicated increased eating frequency as a good strategy for weight loss and weight control (6, 7), while more recent studies have shown the opposite (4, 5). Consuming food less frequently could lead to weight loss by consuming less unhealthy snacks and therefore consuming less calories. In contrast, an increased eating frequency has been linked to a higher diet quality in studies (18, 19), which could be the reason why an increased eating frequency could be a good strategy for weight loss. That participants in our study were all overweight and somewhat homogenous might explain why no association between BMI and eating pattern was revealed.

Obesity is an established risk factor for hypertension. There are, however, few studies reporting on associations between eating patterns and blood pressure. We found that participants eating two prepared meals per day rather than one prepared meal per day had lower systolic blood pressure in crude analyses, but no association remained after adjustment for age and sex. We have so far not been able to find any similar studies linking blood pressure to number of meals with prepared food. Two studies found a link between a higher eating frequency and lower systolic and diastolic blood pressures (20, 21). Other studies have found no associations between eating frequency and the prevalence of hypertension (16, 22). The timing of main meals in relation to blood pressure was examined in one study conducted in 2019 (16). The results revealed that a ‘later’ lunch (after 1 p.m.) compared to a ‘conventional’ lunch (12–1 p.m.) was associated with a higher blood pressure in Australian woman. The reasons behind a potential association between meal frequency and timing of meals and blood pressure are not known. However, there are studies indicating that meal frequency is a possible determinant of diet quality and nutrient intake, and that a greater meal frequency is associated with a better diet quality (18, 19). This suggests that overall diet quality and nutrient intake could play a role in preventing hypertension, rather than the actual eating patterns themselves playing a role. Restricting eating to a shorter time window over the day is another potential eating pattern that might generate health benefits (23). It is apparent that more studies on eating patterns and the relationship with weight control, health, and general anthropometric measurements are needed. Further studies could facilitate for and strengthen the knowledge base for dietary advice focusing on meal order and eating patterns in addition to dietary quality and food content. As there is a large diversity in food preferences, compliance is often difficult when detailed dietary advice is delivered in healthcare.

Older participants ate less prepared meals and snacked more than younger participants. This could indicate a decline in food intake as snacks on a general level are smaller and perhaps often less nutritious than prepared meals. Several studies have demonstrated a decline in food intake with increasing age (24, 25). The mean age of the participants in these studies was 72 years (24) and 52 years (25), while the mean age of the participants in our older age group was 62 years; hence, the results might be applicable to our group. The reasons for this age-related decline in food intake are not completely understood; however, there are many different factors that could play a role. A study exploring dietary habits and food choices of older adults in the United Kingdom identified four themes of age-related changes as possible reasons for the decline in food intake with age: being on your own (living alone and cooking for one), access to food (food costs and support), changes related to age (less physical activity, a decline in hunger), and emotions connected to food (eating a varied diet, dieting) (26). Being on your own could lead to a diminished effort to cook and prepare food as you have no one to share the food with and no one to cook for. Many participants in the study reported preparing simpler meals and eating less frequently later in life. This could be an explanation to why the eating patterns in our older group significantly differed from the younger group. However, as most of the participants in the study from 2018 were > 70 years old, it is not sure that the same reasons could be applicable on the older age group in our study (26).

A difference in eating patterns between men and women has been previously reported. Women reported eating three main meals plus at least one snack to a greater degree than men in a study from 2018 on eating patterns of US adults (27). Both the number of snacks per day and the number of meals per day were higher among women than in men. Also, to be noted is that the American adults reported eating a total of five times per day in general, including two snack episodes daily, a pattern resembling that found in our study. However, as there is no general definition of meals and meal size, a comparison between studies and countries is uncertain.

The strength of our study is the high participation rate and the low internal non-response. The only internal missing data (*n* = 7) were for the item self-rated eating habits. Anthropometrics were taken in a standardized way by experienced nurses, and the questionnaires were completed on location during the health examination. This allowed the participants to be able to ask questions if something was unclear and to ensure that measurements were taken correctly. Carrying out the data collection in this way is considered as a strength. As the study was conducted on a culturally homogenous group of Swedish adults with overweight and obesity, mainly women, it is not possible to generalize conclusions to the general population. However, the fact that the study group was homogenous can also be considered as a strength as the results can be specifically applicable for middle-aged overweight women wanting to lose weight.

The most common method used to assess diet and/or eating patterns on individuals or at a group level is food frequency questionnaires (28). We used a self-invented open matrix question, where answers were interpreted and categorized according to defined categories. This procedure is innovative, and it was perceived easy to understand and answer by the participants during data collection and does not restrict answers to predetermined alternatives. However, validation studies are needed to assess the value and benefit of the questioning approach. The data are limited by the great variation in the extent of the participants’ answers to the open matrix question. A similar food quality assessment method has been used in structured interviews, and an interview may allow for more in-depth and equivalent data (29). Another possible weakness was difficulties in interpreting the answers as the participants completed by hand what they eat in a typical day. Two different people trying to interpret the written answers might not perceive the answers equally, which is a potential source of error. The data material was partially difficult to interpret as the participants completed the matrix question with varying accuracy and detail. An estimation is that 15–20% of the data material was difficult to code into the defined categories. An improvement could be to time the intervals of the eating windows, in which the participants completed what they ate on a typical weekday.

Our study has several limitations such as the cross-sectional design, the small sample size, and the lack of data on food and energy content, which are likely to covary with the number of eating occasions. Multiple statistical testing may impact and increases the risk of false positive outcomes, which may overestime the results. This is of minor inportance since the regression models found no associations between studied variables. However, this small study and the study design do not exclude false negative results. Multicollinearity might be limiting but is not considered problematic in our regression model, given that the Variation Inflation Factors (VIFs) were low. The cross-sectional study design does not allow for assessing causal relationships between eating patterns and how they influence anthropometric measurements, such as BMI, waist measurements, and blood pressure. It is a limitation that blood pressure data were assessed at just one occasion although it was measured twice. More studies are needed to evaluate the potential connections between eating pattern and blood pressure, especially as hypertension is one of the major health concerns, and eating patterns is a modifiable lifestyle factor. Approximately 50 percent of the study population were on medications for hypertension or cardiovascular disease (17). Future analysis of the follow-up trial data will enable prospective analysis. Diet, food content, eating patterns, meal habits, and anthropometrics are closely interconnected to one another, which makes it a complex topic to study, and potential interactions need to be considered. Identifing valid and reliable measurement instruments and methods that are both clinically relevant and practically feasible while ensuring high response rates in the study of dietary factors and health presents a major challenge. There is evidence that it is common to state healthier eating habits than one actually has, which can make results misleading if the information reported is not correct (30).

## Conclusions

The findings of our study demonstrate that the predominant eating pattern among Swedish adults with overweight and obesity was a meal structure encompassing breakfast, lunch, and dinner as prepared meals and two snacks. Older participants, aged 55 years and above, reported a higher snack intake and lower consumption of prepared meals compared to their younger counterparts. Sex differences were observed, with women having more daily eating occasions compared to men. However, no associations were found between eating patterns and anthropometrics or between eating patterns and blood pressure after adjustment for age and sex. Further research on whether and how eating patterns affect anthropometrics and blood pressure is warranted as it might give future insights valuable for health counseling.

## Supplementary Material




