# Supplementary material for: Eating patterns in relation to anthropometrics and blood pressure among adults with overweight and obesity – a cross-sectional study
Source: Ups J Med Sci. 2025 Jul 14;130:10.48101/ujms.v130.12227. doi: 10.48101/ujms.v130.12227 (PMC12320924; doi:10.48101/ujms.v130.12227)
Supplement: Supplementary file 1 [file UJMS-130-12227-s1.pdf]

Survey for participants in the research project "Dare to feel full"

Date you fill in the survey *(year, month, day)* ________________________

1. What year were you born? ____________
(answer with four digits)

2. Are you male or female?

☐ Male ☐ Female

3. What is your main occupation at the moment?

☐ Working full time (or more)
☐ Working part-time
☐ Student
☐ Sick leave (for more than 3 months)
☐ Parental leave
☐ Retired (sick, contract, age-, early-)
☐ Other__________________________

4. If you have a job, what do you do?

____

5. What is your highest formal education?

☐ Elementary school
☐ High school
☐ College/University
☐ Other____

6. What is your current living situation?

☐ Living alone
☐ Living with another person (child and/or adult) fully or partially
☐ Other____

7. How do you generally assess your current eating habits?

- ☐ Very good
- ☐ Good
- ☐ Bad
- ☐ Very bad

8. What do you eat and drink on a regular weekday? *Write down everything you put in your mouth during the day. Specify type of food/drink e.g. 4 cheese sandwiches, 8 meatballs, 3 large potatoes, 2dl sauce, 1 tbsp jelly and peas, 1 apple, 200g chocolate cake, 100 g candy, 2 glasses of juice, 1 strong beer, coffee with or without sugar/milk/cream etc.*

**Time**	**Eat**	**Drink**
**Morning**		
**Mid-morning**		
**Midday**		
**Afternoon**		
**Evening**		
**Night**		

9. What are your tobacco habits? *(multiple options possible)*

- ☐ I have never been a smoker or snus user
- ☐ I quit smoking or snus ______months ago
- ☐ I smoke or use snus, but not daily
- ☐ I smoke daily, __________cigarettes per day
- ☐ I use snus daily, _________cans per week
- ☐ Other___________________________

10. What are your alcohol habits?
The picture shows examples of what is meant by standard glasses.

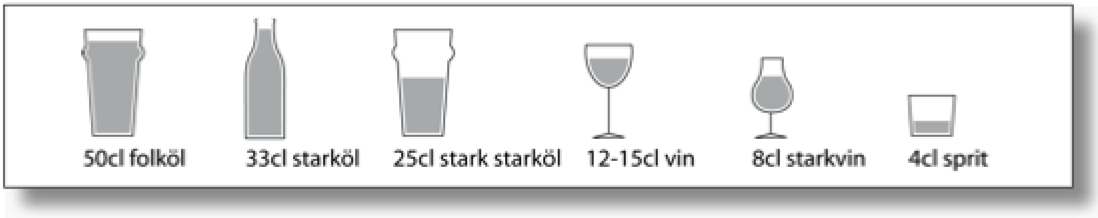

How many standard glasses do you drink in a regular week? *(You can think over a whole month and make an average.)*

- ☐ Do not use alcohol, is teetotaler
- ☐ Drink less than 1 standard glass per week
- ☐ 1–4 standard glasses per week
- ☐ 5–9 standard glasses per week
- ☐ 10–14 standard glasses per week
- ☐ 15 or more standard glasses per week

11. How often do you as a woman drink 4 standard glasses and you as a man drink 5 standard glasses or more at one and the same occasion?

- ☐ Never
- ☐ Less often than 1 time per month
- ☐ Every month
- ☐ Every week

12. What is your physical activity in your current work/main occupation? *(Choose the option that best fits.)*

- ☐ Sedentary
- ☐ Standing
- ☐ Lighter mobile work
- ☐ Heavier mobile work
- ☐ Heavy physical work

13. What is your average physical activity in your free time?

- ☐ Sedentary leisure time
- ☐ Light/moderate exercise in leisure time (do not need to shower afterwards)
- ☐ Moderate, regular exercise in leisure time
- ☐ Regular more intense exercise and training

14. With the help of the following questions, you can try to estimate how satisfied you are with your situation in various respects. (*Mark with a ring around a number for each question.)*

	Very Bad			→			Excellent Could not be better!
1. Work situation	1	2	3	4	5	6	7
2. Economy	1	2	3	4	5	6	7
3. Housing	1	2	3	4	5	6	7
4. Home and family situation	1	2	3	4	5	6	7
5. Appetite	1	2	3	4	5	6	7
6. Sleep	1	2	3	4	5	6	7
7. Memory	1	2	3	4	5	6	7
8. Physical fitness	1	2	3	4	5	6	7
9. Mood	1	2	3	4	5	6	7
10. Stamina	1	2	3	4	5	6	7
11. Energy	1	2	3	4	5	6	7
12. Patience	1	2	3	4	5	6	7
13. Self-confidence	1	2	3	4	5	6	7
14. Health	1	2	3	4	5	6	7

15. Do you take any medication (tablets, injections, patches, nasal spray or other)? ☐

☐ No

☐ Yes, take medication regularly/daily

☐ Yes, use medication as needed *(more than just on occasional occasions)*

If yes, write which medicines it is. If you do not know the name of the medicine, write why you take it (e.g. for blood pressure, painkillers).

____

____

____

____
