# Supplementary material for: Eating patterns in relation to anthropometrics and blood pressure among adults with overweight and obesity – a cross-sectional study
Source: Ups J Med Sci. 2025 Jul 14;130:10.48101/ujms.v130.12227. doi: 10.48101/ujms.v130.12227 (PMC12320924; doi:10.48101/ujms.v130.12227)
Supplement: Supplementary file 2 [file UJMS-130-12227-s2.pdf]

Supplementary table 1. Regression analysis. Dependent variables: BMI, waist circumference, systolic and diastolic blood pressure. Non-standardized b-coefficients, standard error (se) and Variation Inflation Factor [VIF] within brackets.

Modell	BMI (kg/m^2)	Waist (cm)	Systolic blood pressure (mm Hg)	Diastolic blood pressure (mm Hg)
[a]Number of eating occasions/day	0.23^{NS} (1.0)[1.6]	0.00^{NS} (2.3)[1.6]	-2.29^{NS} (4.0)[1.6]	-1.71^{NS} (2.1)[1.6]
[b]Number of snacks/day	-0.30^{NS} (0.7)[1.7]	-0.45^{NS} (1.6)[1.7]	1.88^{NS} (2.8)[1.7]	0.17^{NS} (1.5)[1.7]
[c]Number of meals with prepared food/day	0.19^{NS} (0.5)[1.3]	-0.82^{NS} (1.3)[1.3]	-4.03^{NS} (2.2)[1.2]	1.73^{NS} (1.2)[1.2]
[d]Self-reported eating habits	0.28^{NS} (0.5)[1.1]	-0.60^{NS} (1.1)[1.1]	0.12^{NS} (2.0)[1.1]	1.56^{NS} (1.1)[1.1]
Sex (male/female)	4.25^{NS} (0.6)[1.3]	-12.80*** (1.4)[1.1]	-3.41^{NS} (3.0)[1.7]	-0.38^{NS} (1.6)[1.7]
Age (years)	-0.15*** (0.0)[1.3]	0.31*** (0.1)[1.4]	0.57*** (0.1)[1.4]	-0.20** (0.1)[1.5]
BMI (kg/m^2)		1.80^{NS} (0.1)[1.1]	0.73* (0.3)[3.0]	0.00^{NS} (0.2)[2.7]
Waist (cm)	0.32*** (0.0)[1.3]		-0.17^{NS} (0.1)[3.0]	0.02^{NS} (0.1)[3.0]
Systolic blood pressure (mm Hg)	0.04* (0.0)[1.8]	-0.06^{NS} (0.1)[1.9]		0.33*** (0.0)[1.2]
Diastolic blood pressure (mm Hg)	0.00^{NS} (0.0)[1.7]	0.02^{NS} (0.1)[1.7]	1.14*** (0.1)[1.1]	
Intercept	-0.93^{NS} (0.0)	56.35*** (8.4)	13.87^{NS} (16.6)	47.32*** (8.05)
F Anova	29.1***	35.1***	15.6***	12.9***
N	165	165	165	165

R^2 (adjusted)	0.61	0.65	0.44	0.39

***=p<0.001 **=p<0.01 *p<0.05

[a]Number of eating occasions/day: $\leq$ 3 meals/day or > 3 meals/day.
[b]Number of snacks/day: 0-1 snack/day or >1 snack/day.
[c]Number of meals with prepared food/day: 1 prepared meal or 2 prepared meals.
[d]Self-reported eating habits: Good or bad.
